# Machine learning-based models to predict one-year mortality among Chinese older patients with coronary artery disease combined with impaired glucose tolerance or diabetes mellitus

**DOI:** 10.1186/s12933-023-01854-z

**Published:** 2023-06-14

**Authors:** Yan Li, Lixun Guan, Chaoxue Ning, Pei Zhang, Yali Zhao, Qiong Liu, Ping Ping, Shihui Fu

**Affiliations:** 1 Department of Endocrinology, People’s Hospital of Macheng City, Hubei, China; 2 Hematology Department, Hainan Hospital of Chinese People’s Liberation Army General Hospital, Sanya, China; 3 Central Laboratory, Hainan Hospital of Chinese People’s Liberation Army General Hospital, Sanya, China; 4 School of Life Science, Beijing Institute of Technology, Beijing, China; 5 Medical Care Center, Hainan Hospital of Chinese People’s Liberation Army General Hospital, Sanya, China; 6 General Station for Drug and Instrument Supervision and Control, Joint Logistic Support Force of Chinese People’s Liberation Army, Beijing, China; 7 Department of Cardiology, Hainan Hospital of Chinese People’s Liberation Army General Hospital, Sanya, China; 8 Department of Geriatric Cardiology, Chinese People’s Liberation Army General Hospital, Beijing, China

**Keywords:** Coronary artery disease, Diabetes mellitus, Impaired glucose tolerance, Machine learning techniques, Older patients, One-year mortality

## Abstract

**Purpose:**

An accurate prediction of survival prognosis is beneficial to guide clinical decision-making. This prospective study aimed to develop a model to predict one-year mortality among older patients with coronary artery disease (CAD) combined with impaired glucose tolerance (IGT) or diabetes mellitus (DM) using machine learning techniques.

**Methods:**

A total of 451 patients with CAD combined with IGT and DM were finally enrolled, and those patients randomly split 70:30 into training cohort (n = 308) and validation cohort (n = 143).

**Results:**

The one-year mortality was 26.83%. The least absolute shrinkage and selection operator (LASSO) method and ten-fold cross-validation identified that seven characteristics were significantly associated with one-year mortality with creatine, N-terminal pro-B-type natriuretic peptide (NT-proBNP), and chronic heart failure being risk factors and hemoglobin, high density lipoprotein cholesterol, albumin, and statins being protective factors. The gradient boosting machine model outperformed other models in terms of Brier score (0.114) and area under the curve (0.836). The gradient boosting machine model also showed favorable calibration and clinical usefulness based on calibration curve and clinical decision curve. The Shapley Additive exPlanations (SHAP) found that the top three features associated with one-year mortality were NT-proBNP, albumin, and statins. The web-based application could be available at https://starxueshu-online-application1-year-mortality-main-49cye8.streamlitapp.com/.

**Conclusions:**

This study proposes an accurate model to stratify patients with a high risk of one-year mortality. The gradient boosting machine model demonstrates promising prediction performance. Some interventions to affect NT-proBNP and albumin levels, and statins, are beneficial to improve survival outcome among patients with CAD combined with IGT or DM.

**Supplementary Information:**

The online version contains supplementary material available at 10.1186/s12933-023-01854-z.

## Introduction

Coronary artery disease (CAD) is the main cause of mortality and morbidity worldwide, causing over 8 million deaths worldwide each year and a huge burden on social care systems [1]. Asians exhibited greater susceptibility to CAD and diabetes mellitus (DM) compared to Westerners, due to higher levels of central obesity and total adiposity or higher degree of insulin resistance and endothelial dysfunction [2–4]. Notably, China has the world’s largest number of people with DM, accounting for a quarter of the world’s diabetic patients [5]. As a poor prognostic factor, DM is associated with a two- to four-fold increased risk of developing CAD [6, 7]. Compared to non-diabetic subjects, more severe and diffuse coronary atherosclerosis have been found in diabetic subjects [8]. Similarly, patients with DM more likely experience acute myocardial infarction than patients without DM [9]. That is to say, DM induces subsequent cardiovascular diseases and is detrimental to outcomes among patients with CAD. Survival prediction of patients with CAD helps make appropriate therapeutic strategies, which is able to significantly improve the prognosis of those patients. Proper interventions can be performed in advance and prognosis can be improved more effectively if we promptly achieve risk identification of CAD progression [10]. However, there are few studies on the survival risk factors of older patients with CAD combined with impaired glucose tolerance (IGT) or DM.

Machine learning provides researchers with powerful statistical methods to intercept associations between patient’s features and outcomes, allowing for objective integration of data to predict clinical prognosis. It has been demonstrated to be highly effective methods for prognostic prediction and decision making in plenty of analysis of diverse and massive electronic health record data [11, 12]. Prognostic prediction models have been developed in terms of different machine learning algorithms, and prediction performance of these models was superior to traditional regression-based heart disease prediction [13–15]. Recent systematic review identified that the best predictive model for cardiovascular complications in diabetic patients was neural network, followed by gradient boosting machine model [16]. However, there is still a lack of studies on machine learning for prognostic prediction specifically among older patients with CAD and IGT or DM. Therefore, the purpose of the study was to develop a model to predict one-year mortality among older patients with CAD combined with IGT or DM using machine learning techniques. The logistic regression model, and three machine learning algorithms, including gradient boosting machine model, random forest, and decision tree, were introduced in the study. In addition, a web-based calculator was developed to present optimal model and promote clinical application.

## Methods

### Design and patients

**Figure 1:**
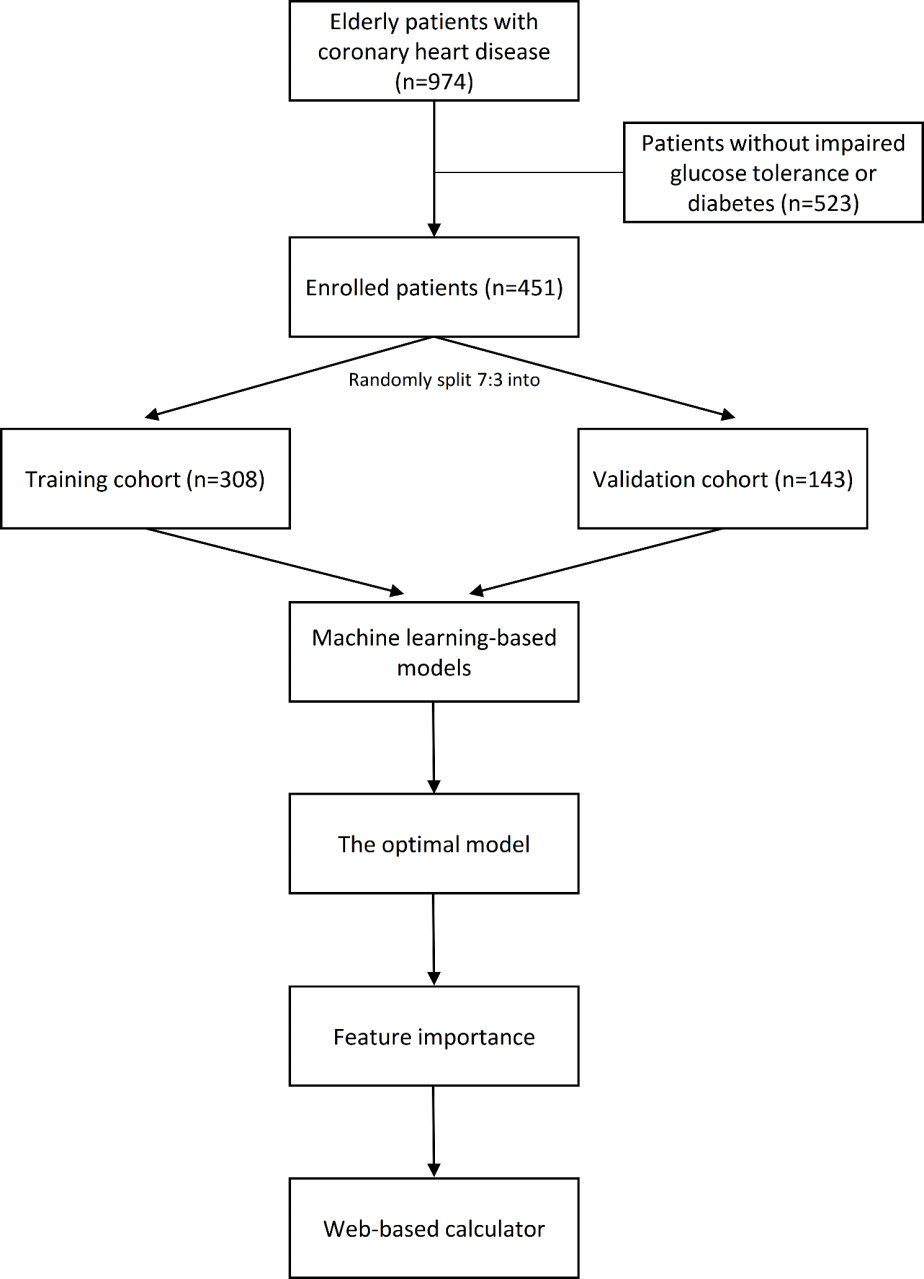
Flowchart outlining patient’s enrollment and study design.

This prospective study analyzed 974 older patients with CAD admitted in the Department of Geriatric Cardiology, Chinese people’s Liberation Army (PLA) General Hospital. Patients were included from October, 2007 to July, 2011 if they (1) aged above 60 years, (2) diagnosed with CAD, and (3) had IGT or DM. Figure 1 shows the flowchart outlining patient’s enrollment and study design. Chinese PLA General Hospital was their designated hospital and had comprehensive medical treatment and final death records, which makes it easier for us to track them for long-term effectively and accurately judge the end point. A total of 451 patients with CAD combined with IGT and DM were finally enrolled, and those patients randomly split 70:30 into training cohort (n = 308) and validation cohort (n = 143). The randomization of patients was achieved using computer. The training cohort was used to train and optimize models using the logistic regression model and three machine learning algorithms, and the validation cohort was used to test prediction performance of these models. This study protocol was approved by the Ethics Committee of Chinese People’s Liberation Army General Hospital (Beijing, China) and in accordance with the Helsinki Declaration of 1975 (as revised in 1983).

## Definition and outcome

According to the guidelines of the American College of Cardiology (ACC)/American Heart Association (AHA)/European College of Cardiology (ESC), the diagnosis of CAD was made by chief physicians based on clinical histories, angina symptoms, cardiac markers, and specific examinations, including electrocardiogram (rest and exercise), echocardiography, radionuclide imaging, computed tomography, and coronary angiography [17, 18]. IGT was defined as 2-h glucose ≥ 7.8mmol/l after a 75-g oral glucose tolerance test (OGTT) [19]. DM was defined as patients with fasting glucose ≥ 7.0mmol/l, OGTT 2-h glucose ≥ 11.1mmol/l, or oral hypoglycemic drugs or insulin [20]. The median interval of follow-up was 5.06 years. Due to the failure of multiple organs in the older population and the priority of all-cause mortality in the outcome study, the primary outcome of the present study was all-cause mortality. One-year mortality was defined as patients died within one year after discharge. Mortality was determined by telephone interviews and medical records, including legal documents of dead time, place and others.

## Data and variables

The present study collected patient’s (1) demographics, including age, gender, current smoker, body mass index (BMI, kg/m^2^), and heart rate, (2) laboratory examination, including glucose (mmol/L), hemoglobin (g/dL), high density lipoprotein cholesterol (HDL-C, mmol/L), low density lipoprotein cholesterol (LDL-C, mmol/L), albumin (g/dL), serum creatinine (µmol/L), uric acid (µmol/L), N-terminal pro-B-type natriuretic peptide (NT-proBNP, pg/ml), (3) comorbidities, including hypertension, chronic heart failure (CHF), and chronic kidney disease (CKD), (4) drugs used during hospitalization, including aspirin, clopidogrel, beta receptor blockers, calcium channel blockers (CCB), nitrates, angiotensin converting enzyme inhibitor (ACEI) or angiotensin receptor blocker (ARB), and statins. All information was obtained and preserved by trained researchers. To verify the accuracy of the results, other independent researchers performed logistical check and data re-evaluation. Blood tests were conducted at the central laboratory in the Department of Biochemistry, Chinese PLA General Hospital.

## Modelling and validation

To develop prediction model for one-year mortality among those patients, the logistic regression model and three machine learning models including gradient boosting machine model, random forest, and decision tree were used to develop prediction models in the training group. The least absolute shrinkage and selection operator (LASSO) method combined with ten-fold cross-validation was used to screen characteristics associated with one-year mortality, and significant variables were used as inputs to train and optimize models. Random hyper-parameter search was performed to determine the model parameters after applying 5-fold cross-validation and 100 iterations of bootstrapping procedures to achieve the best area under the curve (AUC). Patients in the validation group were used to assess predictive effectiveness of models using the Brier score, AUC, calibration curve, and decision curve. The following formula was used to calculate the Brier score.

$$Brier Score= \frac{1}{N}\sum _{i=1}^{n}{({p}_{i}-{o}_{i})}^{2}$$

In the formula, $N$ is the sample size, ${p}_{i}$ is the predicted probability of death within one year, and ${o}_{i}$ is the actual probability of death within one year (“No” vs. “Yes”).

Shaley Additive exPlanation (SHAP) was performed to determine feature contributions using the following formula.

$$g\left({z}^{{\prime }}\right)={\varphi }_{0}+\sum _{j=1}^{M}{\varphi }_{j}{{Z}^{{\prime }}}_{j}$$

In the formula, $g$ is the interpretation model, $M$ is the number of inputted parameters, ${\varphi }_{0}$ is a constant, and ${\varphi }_{j}$ is the attribution value (Shapley value) of each model parameter.

## Developed web calculator

A web-based calculator for predicting one-year mortality among those patients was developed using the “Streamlit” (https://share.streamlit.io/) application in terms of the optimal model. For friendly use of the web calculator, the present study deployed three panels, including the panel for choosing and filling model parameters, the panel for getting predicted one-year mortality, and the panel for model introduction.

## Statistics and implementation

Categorical variables were presented as proportion, and continuous variables that were not normally distributed were presented as median and interquartile range (IQR). A comparison of characteristics was performed between patients with and without one-year mortality using Wilcoxon rank tests for not normally distributed continuous variables and using Chi-square or continuous adjusted Chi-square tests for categorical variables. Reclassification of patients was performed according to the best cut-off value of the optimal model, and two risk (high-risk vs. low-risk) groups were categorized in the validation group. Traditional statistical analyses were performed using R programming language (version 4.1, http://www.R-project.org). A P value of less than 0.05 was considered as significant (two-tailed). Machine learning modelling and interpretation were performed in an open-source web application of *Jupyter Notebook* in which authors are able to use Python language (version 3.9).

## Results

### Patient’s basic characteristics

The median age of the entire cohort was 86.00 [82.00, 89.00] years (Table 1). The majority of patients were male (89.6%). The comorbidity burden was relatively heavy, because 87.1% patients had hypertension, 85.1% patients had DM, 71.0% had chronic CAD, 31.0% had CHF, and 36.6% had CKD. Drugs used during hospitalization was common among those patients. To elaborate, aspirin, clopidogrel, beta receptor blocker, CCB, nitrates, ACEI/ARB and statins were administrated among 46.3%, 62.7%, 73.2%, 71.4%, 85.4%, 57.6% and 67.2% patients, respectively. The one-year mortality was 26.83% among all patients. Subgroup analysis was performed to compare clinical characteristics between patients with and without one-year mortality. Patients who died within one year tended to have an older age, a lower BMI, and a higher heart rate (P < 0.05 for all). Meanwhile, patients who were dead within one year had higher glucose, serum creatinine, uric acid and NT-proBNP levels, and lower hemoglobin, HDL-C, LDL-C and albumin levels (P < 0.05 for all). In addition, patients with one-year mortality tended to have an elevated proportion of comorbidities such as more DM, CHF and CKD (P < 0.05 for all). Regarding administration of drugs during hospitalization, patients with one-year mortality had significant less aspirin, clopidogrel, ACEI/ARB, and statins (P < 0.05 for all).

**Table 1:** Patient’s demographics, tests, comorbidities, and drugs

Characteristics	Whole	One-year mortality		P	Cohort		P
Groups	Patients	No	Yes		Training	Validation	
n	451	330	121		308	143	
**Demographics**							
Age (median [IQR], years)	86.00 [82.00, 89.00]	85.00 [81.00, 89.00]	88.00 [85.00, 91.00]	< 0.001	86.00 [82.00, 89.00]	86.00 [82.00, 89.00]	0.655
Gender (male/female, %)	404/47 (89.6/10.4)	297/33 (90.0/10.0)	107/14 (88.4/11.6)	0.757	273/35 (88.6/11.4)	131/12 (91.6/8.4)	0.426
Current smoker (no/yes, %)	426/25 (94.5/5.5)	312/18 (94.5/5.5)	114/7 (94.2/5.8)	1.000	289/19 (93.8/6.2)	137/6 (95.8/4.2)	0.528
BMI (median [IQR], kg/m^2^)	24.49 [22.36, 26.92]	24.83 [22.84, 26.91]	23.63 [21.22, 27.34]	0.010	24.48 [22.13, 26.74]	24.68 [22.72, 27.14]	0.402
Heart rate (median [IQR])	72.00 [65.00, 80.00]	71.00 [64.00, 79.00]	75.00 [67.00, 85.00]	0.002	70.00 [64.00, 80.00]	73.00 [66.00, 80.00]	0.054
**Tests**							
Glucose (median [IQR])	5.70 [4.99, 6.78]	5.60 [4.96, 6.59]	6.10 [5.10, 7.80]	0.006	5.70 [4.98, 7.01]	5.68 [5.01, 6.64]	0.661
Hemoglobin (median [IQR], g/dL)	123.00 [109.00, 135.00]	126.00 [116.00, 138.00]	107.00 [94.00, 122.00]	< 0.001	121.00 [107.00, 134.00]	124.00 [112.50, 136.00]	0.107
HDL-C (median [IQR], mmol/L)	1.01 [0.85, 1.20]	1.05 [0.88, 1.23]	0.88 [0.67, 1.11]	< 0.001	1.01 [0.86, 1.20]	1.04 [0.84, 1.21]	0.561
LDL-C (median [IQR], mmol/L)	2.07 [1.58, 2.55]	2.11 [1.65, 2.57]	1.90 [1.34, 2.51]	0.010	2.08 [1.61, 2.53]	2.05 [1.48, 2.66]	0.731
Albumin (median [IQR], g/dL)	38.50 [35.20, 40.90]	39.20 [37.00, 41.40]	34.40 [31.60, 37.60]	< 0.001	38.35 [35.00, 40.62]	38.90 [35.95, 41.05]	0.159
Serum creatinine (median [IQR], µmol/L)	89.00 [72.75, 121.50]	87.00 [73.00, 111.53]	104.30 [71.80, 156.40]	0.003	89.00 [72.00, 121.25]	91.20 [74.00, 122.00]	0.613
Uric acid (median [IQR], µmol/L)	335.50 [249.00, 415.95]	328.80 [253.35, 398.20]	364.00 [231.30, 489.10]	0.020	340.75 [252.68, 417.22]	329.70 [247.60, 413.80]	0.715
NT-proBNP (median [IQR], pg/ml)	447.10 [172.40, 1762.00]	296.10 [138.70, 906.02]	2042.80 [690.60, 5848.30]	< 0.001	456.25 [177.52, 1762.95]	408.10 [148.35, 1738.10]	0.589
**Comorbidities**							
Hypertension (no/yes, %)	58/393 (12.9/87.1)	41/289 (12.4/87.6)	17/104 (14.0/86.0)	0.766	38/270 (12.3/87.7)	20/123 (14.0/86.0)	0.737
DM (no/yes, %)	67/384 (14.9/85.1)	58/272 (17.6/82.4)	9/112 (7.4/92.6)	0.011	41/267 (13.3/86.7)	26/117 (18.2/81.8)	0.226
CAD (chronic/acute, %)	320/131 (71.0/29.0)	236/94 (71.5/28.5)	84/37 (69.4/30.6)	0.751	217/91 (70.5/29.5)	103/40 (72.0/28.0)	0.817
CHF (no/yes, %)	311/140 (69.0/31.0)	254/76 (77.0/23.0)	57/64 (47.1/52.9)	< 0.001	209/99 (67.9/32.1)	102/41 (71.3/28.7)	0.527
CKD (no/yes, %)	286/165 (63.4/36.6)	228/102 (69.1/30.9)	58/63 (47.9/52.1)	< 0.001	194/114 (63.0/37.0)	92/51 (64.3/35.7)	0.864
**Drugs**							
Aspirin (no/yes, %)	242/209 (53.7/46.3)	165/165 (50.0/50.0)	77/44 (63.6/36.4)	0.014	166/142 (53.9/46.1)	76/67 (53.1/46.9)	0.962
Clopidogrel (no/yes, %)	168/283 (37.3/62.7)	105/225 (31.8/68.2)	63/58 (52.1/47.9)	< 0.001	120/188 (39.0/61.0)	48/95 (33.6/66.4)	0.318
Beta receptor blocker (no/yes, %)	121/330 (26.8/73.2)	94/236 (28.5/71.5)	27/94 (22.3/77.7)	0.234	90/218 (29.2/70.8)	31/112 (21.7/78.3)	0.117
CCB (no/yes, %)	129/322 (28.6/71.4)	88/242 (26.7/73.3)	41/80 (33.9/66.1)	0.166	81/227 (26.3/73.7)	48/95 (33.6/66.4)	0.140
Nitrates (no/yes, %)	66/385 (14.6/85.4)	50/280 (15.2/84.8)	16/105 (13.2/86.8)	0.717	40/268 (13.0/87.0)	26/117 (18.2/81.8)	0.190
ACEI/ARB (no/yes, %)	191/260 (42.4/57.6)	125/205 (37.9/62.1)	66/55 (54.5/45.5)	0.002	134/174 (43.5/56.5)	57/86 (39.9/60.1)	0.531
Statins (no/yes, %)	148/303 (32.8/67.2)	80/250 (24.2/75.8)	68/53 (56.2/43.8)	< 0.001	101/207 (32.8/67.2)	47/96 (32.9/67.1)	1.000
Death within one year (no/yes, %)					217/91 (70.5/29.5)	113/30 (79.0/21.0)	0.072
IQR, interquartile range; BMI, body mass index; HDL-C, high density lipoprotein cholesterol; LDL-C, low density lipoprotein cholesterol; NT-proBNP, N-terminal pro-B-type natriuretic peptide; DM, diabetes mellitus; CAD, Coronary artery disease; CHF: chronic heart failure; CKD, chronic kidney disease; CCB, calcium channel blockers; ACEI, angiotensin converting enzyme inhibitor; ARB, angiotensin receptor blocker.							

## Development of models

**Figure 2:**
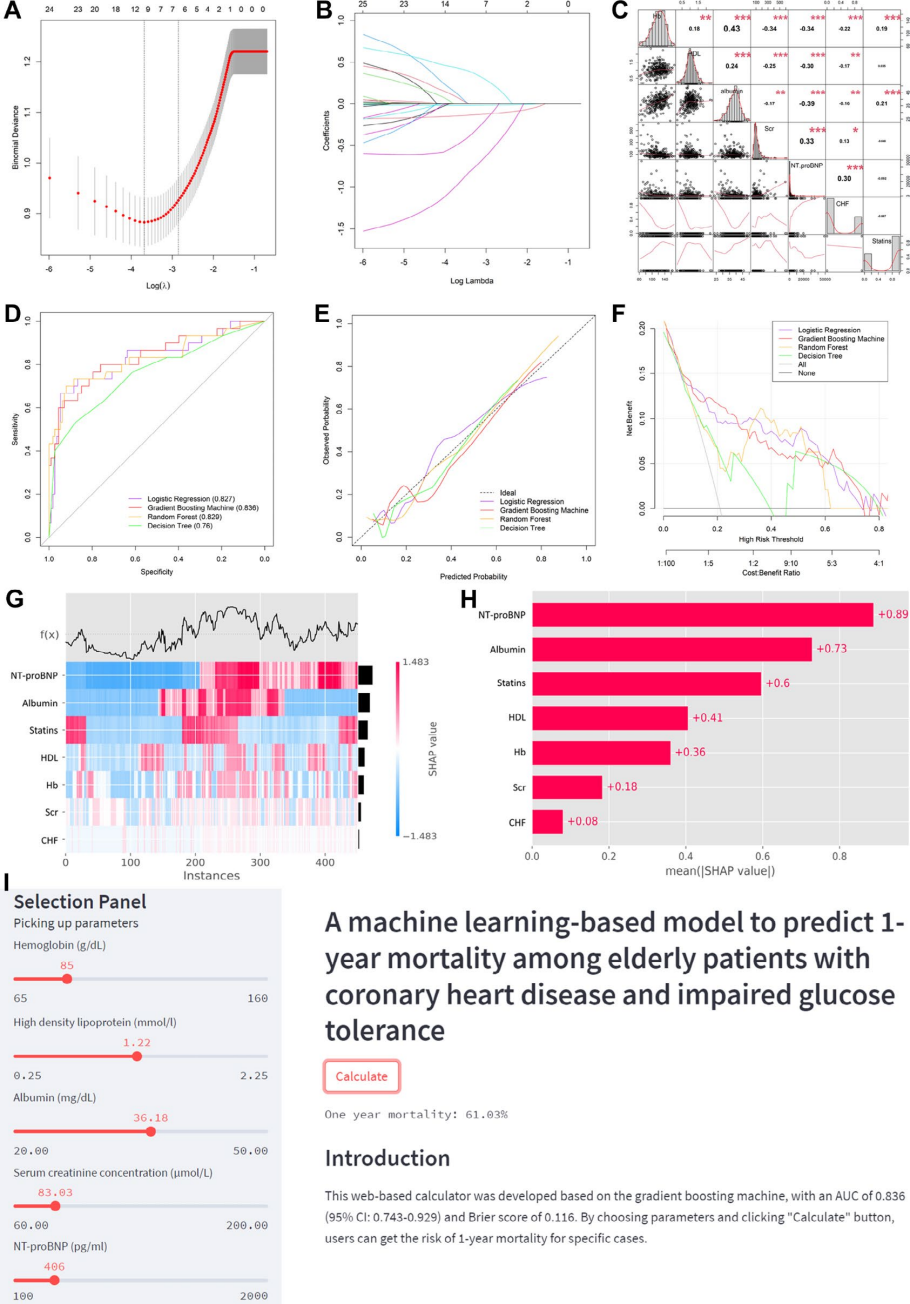
A: The least absolute shrinkage and selection operator (LASSO) coefficient profiles of all variables; B: Selection of appropriate parameters; C: Correlation coefficients between clinical characteristics; D: Overall performance of each model; E: Calibration curve of each model; F: Clinical decision curve analysis of each model; G: The heatmap of SHAP value; H: Analysis of feature importance based on SHAP summary plot; I: The screenshot of online calculator.

According to the least absolute shrinkage and selection operator (LASSO) method and ten-fold cross-validation (Fig. 2A B), seven clinical characteristics were identified to be significantly associated with one-year mortality, and these characteristics included hemoglobin, HDL-C, albumin, serum creatinine, NT-proBNP, CHF, and statins. In addition, serum creatinine, NT-proBNP, and CHF were risk factors based on the LASSO method, with hemoglobin, HDL-C, albumin, and statins being protective factors. Correlation coefficients between those clinical characteristics were calculated and presented as a correlation matrix (Fig. 2C). It demonstrated that no serious collinearity existed because all the correlation coefficients were below 0.80. Thus, the seven clinical characteristics were served as model predictors to establish models using the logistic regression and three machine learning models. The optimal full super-parameters for each model were obtained after training and optimizing models (Table 2).

**Table 2:** Full super-parameters of techniques

Logistic Regression:
LogisticRegression (C = 100, random_state = 42)
Gradient Boosting:
GradientBoostingClassifier (max_depth = 25, max_features=’log2’, min_samples_leaf = 64, min_samples_split = 191, n_estimators = 101, random_state = 42)
Decision Tree:
DecisionTreeClassifier(max_depth = 8, max_features=’auto’, min_samples_leaf = 28, min_samples_split = 22, random_state = 42)
Random Forest:
RandomForestClassifier(max_depth = 33, max_features=’log2’, min_samples_leaf = 34, min_samples_split = 143, n_estimators = 21, random_state = 42)

## Validation of models

As for the overall performance of each model, the Brier score was 0.116 for logistic regression model, 0.114 for gradient boosting machine model, 0.143 for decision tree model, and 0.126 for random forest model. Corresponding AUC was 0.827 [95% confidence interval (CI): 0.731–0.924], 0.836 (95% CI: 0.743–0.929), 0.760 (95% CI: 0.652–0.869), and 0.829 (95% CI: 0.728–0.930), respectively (Fig. 2D). The above findings elucidated that the gradient boosting machine model was the optimal model in terms of the Brier score and AUC. Calibration curve showed that all models, in particular the gradient boosting machine model, had favorable calibration (Fig. 2E). Clinical decision curve analysis showed that the gradient boosting machine model and the logistic regression model both showed favorable clinical usefulness (Fig. 2F). Based on the gradient boosting machine model, the study further analyzed the feature importance for significant clinical characteristics in the whole population. The individual SHAP value and the mean SHAP value both found that the top three features associated with one-year mortality were NT-proBNP, albumin, and statins (Fig. 2G H).

## Optimal web-based calculator

As the gradient boosting machine model was the optimal model in the study, we further developed a web-based calculator for predicting one-year mortality using this model in the “Streamlit” application (https://share.streamlit.io/). In the calculator, the present study deployed a panel for choosing model parameters, a panel for obtaining predicted one-year mortality, and a panel for introduction of the model (Fig. 2I). This calculator could be available at https://starxueshu-online-application1-year-mortality-main-49cye8.streamlitapp.com/.

Sometimes, if the web-based calculator has gone to sleep (shut down) and can not be opened, users are able to access to it via clicking ‘Yes, get this app back up!’. After about 30 s, the web-based calculator would be accessible.

## Reclassification of patients

Patients were recategorized according to the best cut-off value (26.4%) in the gradient boosting machine model. Patients with a predicted risk of less than 26.4% were categorized into the low-risk group, whereas patients who had a predicted risk of 26.4% or above were categorized into the high-risk group. To be specific, the predicted risk was 7.72% in the low-risk group, and 62.31% in the high-risk group; the actual risk was 7.22% (7/97) in the low-risk group, and 50.00% (23/46) in the high-risk group. It indicated that patients in the high-risk group were near seven times more likely to suffer from one-year mortality after discharge from hospitalization.

## Discussion

The present study developed an accurate model to predict one-year mortality among older patients with CAD and IGT or DM. Three machine learning techniques were introduced for analysis in the study, and the gradient boosting machine model showed the optimal prediction performance as compared to other models. The AUC of the model was up to 0.836, indicating excellent prediction effectiveness. In addition, to encourage clinical use of the model, the study proposed a web-based application, and the application was user-friendly. The LASSO method and ten-fold cross-validation identified that seven clinical characteristics were significantly associated with one-year mortality with creatine, NT-proBNP, and CHF being risk factors and hemoglobin, HDL-C, albumin, and statins used during hospitalization being protective factors. The SHAP found that the top three features associated with one-year mortality were NT-proBNP, albumin, and statins.

In the past two decades, the incidence of DM in China has been on a significant rise. Half of the adults (50.1%) in China are thought to have prediabetes. In other words, they have impaired fasting glucose and/or IGT [21, 22]. In addition, the prognosis of CAD is worse in patients with DM than in nondiabetic patients. IGT is also a risk factor for cardiovascular diseases [23, 24]. Most of the current studies on the prognosis of CAD focus on the analysis of patients’ overall survival and risk factors. Studies on the risk of short-term mortality in older patients with CAD combined with IGT or DM are still rare. The present study developed a new model with the aim of helping clinicians to identify patients at risk of short-term mortality in time for early warning and treatment to improve the survival of older patients with CAD.

In the present study, the LASSO method and ten-fold cross-validation identified that seven clinical characteristics were significantly associated with one-year mortality with creatine, NT-proBNP, and CHF being risk factors and hemoglobin, HDL-C, albumin, and statins used during hospitalization being protective factors. The SHAP found that the top three features associated with one-year mortality were NT-proBNP, albumin, and statins. To elaborate, NT-proBNP is a well-known biomarker for CAD, and it may facilitate early diagnosis of heart failure and stratification of cardiac risk. NT-proBNP has been reported to be a significant predictor of mortality for cardiovascular diseases [25]. Elevated NT-proBNP levels are associated with left ventricular insufficiency and poorer clinical prognosis in patients with heart failure and other related diseases. These observations have since been extended to patients with CAD, where elevated NT-proBNP levels may also be the result of myocardial ischemia [26]. As demonstrated previously, increasing quartiles of NT-proBNP were strongly related to an increase in the odds of one-year mortality [27]. In a study of patients with CAD, NT-proBNP levels were strongly associated with one-year mortality for the fourth versus the first quartile [28].

Serum albumin is an acute phase protein whose serum levels are affected by the nutritional status, inflammatory responses, and fluid status, exerting a protective effect in atherosclerosis via its anti-inflammatory, anti-oxidant, and anti-thrombotic roles [29]. Several studies have revealed that low serum albumin levels predicted adverse outcomes in the general population, as well as in patients with CAD [30]. A previous meta-analysis demonstrated that low serum albumin levels are associated with an increased risk of death, not only in subjects free from cardiovascular diseases, but also in patients who already experienced cardiovascular diseases [31]. These findings suggested that serum albumin could be a potential prognostic biomarker for cardiovascular diseases. Older patients are often at risk for malnutrition, infection, overloaded fluid, and liver and kidney insufficiency, all of which lead to the development of hypoproteinemia and an increased risk of short-term mortality in older patients. The inclusion of serum albumin into the model in our report is benefit for clinicians to identify potential risks in older patients with CAD in a timely manner, thus assisting clinical decision-making and therapy.

It is well-known that stains can improve clinical prognosis in the primary and secondary prevention of CAD. Both abnormal inflammatory responses or abnormal lipid metabolism are risk factors for CAD and DM. Dyslipidemia often accompanies abnormal glucose metabolism. As an essential risk factor for CAD, DM can exacerbate the progression of atherosclerosis, resulting in poor clinical outcomes. Abnormal blood glucose metabolism, including IGT and DM, has become increasingly common. As a key risk factor for CAD, DM can aggravate the progression of atherosclerosis and lead to adverse clinical outcomes [32, 33]. CAD and DM have common abnormal inflammatory response or abnormal lipid metabolism, and glucose metabolism is closely related to lipid metabolism [34]. Therefore, lipid metabolism and its management play an important role in CAD patients with CAD combined with IGT or DM.

As one of the important prognostic markers for patients with CAD, epidemiologic studies have demonstrated inverse relationship between HDL-C levels and CAD occurrence. HDL-C can mediate reverse transport of cholesterol to regulate atherosclerotic process and exhibits anti-inflammatory, anti-oxidant, and anti-thrombotic effects on vascular system. Studies reported that elevated HDL-C levels after treatment with stains can reduced the incidence of death [35]. The seven parameters included in our model are all common clinical indicators, with advantages of accessibility and convenience, which contribute to clinical application and popularization of the model. For high-risk patients, we should intervene in advance to control adverse prognostic factors and reduce death risk of patients with CAD combined with IGT or DM.

Actually speaking, previous studies have developed models to predict survival outcome among patients with CAD using machine learning techniques. For instance, Sherazi et al. [36] developed a machine learning-based one-year mortality prediction model after applying 22 continuous variables, 43 categorical variables, and 4 discrete variables. Although the AUC of the best machine learning model could be up to 0.898, too many variables in the model may restrict clinical application of the model. In addition, web-based calculator was not available in the above study. Ke et al. [10] showed that the gradient boosting decision tree had the optimal performance with the AUC of 0.918, but the model was used to predict the in-hospital mortality among patients with CAD. In the present study, the AUC of our model was up to 0.836, indicating excellent prediction effectiveness. To encourage clinical use of the model, the study also proposed a web-based application, and the application was user-friendly. In clinical settings, users are able to fill parameters of each feature according to patient’s conditions in the panel of selecting parameters, and then the probability of one-year mortality could be presented in the panel of results via submitting all these parameters. Based on the reclassification of patients, patients in the high-risk group were near seven times more likely to suffer from one-year mortality. Therefore, for patients in the high-risk group, effective measures need to be taken early based on the findings of the study, including regulating the levels of hemoglobin, albumin, creatine and NT-proBNP, treating CHF, and administering statins for appropriate patients.

The present study had some drawbacks. For one thing, although this study analyzed up to 26 variables, respiratory infections may have a significant impact on survival outcome and were not included for analysis of this study. For another thing, because the model was not externally validated in the present study, the generalization of the model in other cohorts needs future investigation.

## Conclusions

The present study proposes an accurate model that is able to stratify patients with a high risk of one-year mortality. The gradient boosting machine model demonstrates promising prediction performance. Some interventions to affect NT-proBNP and albumin levels, and statins used during hospitalization, are beneficial to improve survival outcome among patients with CAD combined with IGT or DM.

## Electronic supplementary material

Below is the link to the electronic supplementary material.

Supplementary Material 1

Supplementary Material 2

## Data Availability

All data and material are available under the requirement to the corresponding authors.
